# Emotional Information in News Reporting on Audience Cognitive Processing in the Age of Posttruth: An Electroencephalogram and Functional Connectivity Approach

**DOI:** 10.3389/fpsyg.2021.734147

**Published:** 2021-08-12

**Authors:** Ya Yang, Lichao Xiu, Guoming Yu

**Affiliations:** Lab of Cognitive Neuroscience and Communication, School of Journalism and Communication, Beijing Normal University, Beijing, China

**Keywords:** emotion, posttruth, news reporting, EEGs, wPLI, functional connectivity

## Abstract

The purpose of the present study is to explore how the emotionalized expression of news content in the posttruth era affects the cognitive processing of the audiences. One news that was text-written with two different expression types (emotional expression vs. neutral expression) was adopted as an experiment material in the study, and changes in cortical activity during news reporting reading tasks were examined with electroencephalograms, sampled from nine sites and four channels and analyzed with weighted phase lag index (wPLI) based on brain functional connectivity (FC) method. The results show that emotional discourses caused a stronger cortical brain activity and more robust brain FC (beta oscillations); besides, reading emotionalized expression consumed more attention resources but fewer cognitive resources, which may impede further rational thinking of the audiences.

## Introduction

Posttruth, which was selected as the annual word by Oxford Dictionary in 2016, is defined as that emotion and personal belief that is more effective than objective facts in shaping public opinions (Scheufele and Krause, 2019). The narration of the posttruth, tracing to the description for political events, has been used to serve a particular political purpose manipulating truths and facts with the discourse. Previous studies have shown a commonly shared concern of scholars that the fundamental belief of truths and facts has gradually been faded away in the global scene (Laybats and Tredinnick, 2016; Oxford Dictionaries, 2016; Gewin et al., 2017; Rochlin, 2017).

Posttruth alludes to the recognition of the influence of other factors for the truth rather than a denial of truth. The concept of the posttruth has expanded from the political region to more extensive fields, and it is defined in the process of information receiving the reaction of audiences to all forms of truth (Keane and Wight, 2017). It is emphasized that the analysis of the posttruth should originate from more standpoints of the audiences, involving their individualized interpretation of information from different perspectives and their emotions, experiences, and feelings, which would interrupt their own cognition of the truth (Weeks, 2015). It could be seen that emotional expression is an essential feature of media information in the age of posttruth.

Currently, the critical role of emotions has been realized in studies such as cognitive neuroscience and communication (Meyer et al., 2014; Crilley, 2018), where a number of primary researches have explored the impact of emotionalized expression on the audiences under the context of new media. In particular, emotionalized information could affect the cognition of information of the audiences through emotional contagion, besides their judgment on the usefulness of the information with further influence of their follow-up communication behaviors (Ding et al., 2014; Kramer, 2014; Hong et al., 2016). However, self-report or computational online data analysis involved is more frequently used to measure how emotionalized information would affect the cognition and behavior of the individuals. It is often criticized for lacking accuracy or being too macroscopic, and so on the microlevel, the cognitive processing and emotional experience of the individuals could not be reflected in real time.

In cognitive neuroscience experiments, which could be considered as good open science practices, electroencephalography (EEG) measure has been widely applied, which comprehensively reflects through a record of neural activity in the cerebral cortex the cognitive mechanism of the individual in the information processing. The technology is harmless to humans, and the data recording is facilitated with higher time-resolution, leading to a distinct advantage in the implicit monitoring of real-time information processing of an individual. In the previous research, EEG technology has played an essential role in the emotional experience measurement of the individuals, especially when some subtle emotional stimuli could not be easily perceived subjectively, with different emotion statuses that could be reflected by the fluctuations of EEG signals (Kamarajan et al., 2004; Ohme et al., 2009, 2010; Vecchiato et al., 2011; Cartocci et al., 2016; Chen et al., 2017). By far, many studies to have proved a close relationship between the emotional experience of the individuals and their cognitive processes through EEG experiments.

Emotional cognitive processing, additionally, involves the coordination of multiple brain regions; thus, it is necessary to investigate the functional connectivity (FC) between different EEG channels. FC analysis enables the description of cognitive processes within the human brain to support further stage distinctions of brain activities (Torres-Valencia et al., 2017) and is also widely applied in exploring emotion processing mechanism. For instance, the non-linear connectivity of phase-locking value in alpha, beta, and theta oscillations have been used to study the FC of positive and negative emotions induced by different types of stimuli in the brain network (Dasdemir et al., 2017). Moreover, the weighted phase lag index (wPLI) is an acknowledged and robust estimation to detect alterations of functional brain connectivity at the consciousness level (Imperatori et al., 2019).

Hence, the study has been undertaken with the hypothesis that the emotionalized expression of news content in the posttruth era could affect the cognitive activities of the audiences. One news text that was written with two different expression types (emotional expression vs. neutral expression) was adopted as the experiment material in this study, with the EEG measures to record brain signals in the reading process, and the FC method was used to analyze the FC of EEG signals in different oscillations to observe the influence of emotional discourse on individual cognitive activities.

## Materials and Methods

### Participants

A total of 50 right-handed undergraduates and graduates were recruited and randomly divided into two groups; one experimental group (13 women and 12 men, mean age 22.16 ± 1.91), and one control group (13 women and 12 men, mean age 22.96 ± 2.32). All the participants reported normal or corrected-to-normal vision, had no history of current or past neurological or psychiatric illness, and took no medications known to affect the central nervous system. Their emotional statuses were assessed normal by the Chinese beck anxiety inventory, beck depression inventory, and the positive and negative affect scale (as shown in Table 1). There was no significant difference in the scores of positive and negative emotions [*t* (48) = 1.240, *p* = 0.221; *t* (48) = 0.303, *p* = 0.763] between the two groups before the experiment. They signed consent before the experiments and were paid for their participation.

**TABLE 1:** Age, BAI, BDI, and PANAS scores of the experimental and control group.

	**Experimental group (*N* = 25)**	**Control group (*N* = 25)**
Male	12	12
Female	13	13
Age	22.16 ± 1.91	22.96 ± 2.32
BAI	29.56 ± 5.65	28.04 ± 4.46
BDI	9.00 ± 6.17	7.80 ± 6.44
Positive emotion	29.60 ± 5.46	20.92 ± 6.65
Negative emotion	31.46 ± 5.00	20.29 ± 7.85

### Materials

The two groups were asked to read two news texts (with emotional expression vs. neutral expression). Control group read the news article *The United States Will Begin Compulsory Labeling of Genetically Modified Foods* with 1,369 Chinese characters, concerning the unbiased introduction of the technology of Genetically Modified (GM) foods and food labeling regulation; while the experimental group read the news version which was modified with more emotional expressions and discourses based on the previous one, the original argument was kept unchanged, with 1,343 Chinese characters. All the news materials were printed on A4 papers of size 210 mm × 297 mm.

### Procedure

The participants were instructed to have a 3-min resting state before the experiment, and then they were asked to read the text in detail. After the reading, they were asked to answer six questions related to the versions to deepen the image of the content. After finishing the experiment, they took a 3-min tranquillization to recover the emotion status to benchmark. The reading time was about 4 min, and the total experiment time was about 10 min.

### Recordings and Analysis

The raw EEG data were real-time recorded from Cognionics Quick-30 32 channels amplifier (CGX, San Diego, CA, United States), which is a dry and non-contact wireless bioelectric sensor system, and sampled at 1,000 Hz, with a 0–100 Hz bandpass. The left mastoid electrodes were used as a reference during recording, and a standard average reference was calculated off-line. Artifacts were corrected using independent component analysis method, and the averaged EEG signals were low-pass filtered at 30 Hz and divided by every 2 s, using EEG Lab 14.1.1 software.

The EEG recording sites were F3, Fz, F4, C3, Cz, C4, P3, Pz, and P4. The average power spectral density (PSD), which were extracted from all the sites on four bands of delta (1–4 Hz), theta (4–8 Hz), alpha (8–13 Hz), and beta (13–30 Hz), were analyzed and the PSD values between the two groups were compared with independent *t*-test using SPSS 24.0.

Furthermore, the brain FC neuroscience method was also used to show scale-free current source density (CSD) to investigate the brain activity of the audience in the processing of reading news texts. CSD was extracted from the four bands and then the wPLI index was calculated. Whole-brain connectivity was drawn based on the average wPLI, with independent *t*-test and false discovery rate adjusting, to analyze interregional brain interactions.

## Results

### PSD Results

As presented in Table 2, the scalp EEG PSD values show significant differences between the experimental group and the control group (reading emotional news text vs. neutral news text).

**TABLE 2:** PSD value (δ, θ, α, and β) and *t*-test between experimental group and control group.

**EEG Rhythm Wave**	**Experimental Group (*N* = 25)**	**Control Group (*N* = 25)**	**t**
	(*MSD*)	(*MSD*)	(*df* = 48)
δ	2.7920.686	3.4060.892	−2.728**
θ	0.9390.448	1.3490.592	−2.764**
α	0.1780.417	0.5580.491	−2.952**
β	−0.6600.379	−0.2610.421	−3.518***

***p ≤ 0.001; 0.001 < **p ≤ 0.01.

First, the delta EEG rhythm wave [*t* (48) = −2.728, *p* = 0.009, Cohen’s *d* = −0.614], which related to the complexity of the task (Harmony et al., 1996), shows that in the experimental group the participants encountered a more complex reading task, that is, to say a reading with emotional discourse, leading to more attention resources to be input.

Second, the theta oscillations index [*t* (48) = −2.764, *p* = 0.008, Cohen’s *d* = −0.410], which is related to the arousal and control of working memory (Gevins et al., 1997; Krause et al., 2000; Raghavachari et al., 2001; Deiber et al., 2007), shows that in experimental group the participants had left fewer attention resources for memory.

Third is the alpha EEG power [*t* (48) = −2.952, *p* = 0.005, Cohen’s *d* = −0.381]. The weaker the alpha was (Davidson, 1995; Sarlo et al., 2005), the stronger cortical activity the participants had during the emotional text reading task.

Fourth, it is worthy to note what the beta EEG rhythm oscillation [*t* (48) = −3.518, *p* = 0.001, Cohen’s *d* = −0.399] showed. As the PSD value of beta wave in the experimental group was lower than that of the control group, and the beta wave is related to awareness and cognition (Haenschel et al., 2000; Tibbetts, 2013), it shows that the participants of the emotional text reading group had a weaker cognitive resource input, which could indicate that the news content with emotional expression may hinder deep thinking of the individuals in the process of reading.

### FC Results

In Table 3, the differences in the whole-brain connectivity wPLI on all the bands are presented. Notedly, FC on beta band was significant [*t* (48) = 58.382, *p* = 0.0008, Cohen’s *d* = 17.250], across all sites and conditions, but there was no main significance on other bands [alpha, *t* (48) = 0.521, *p* = 0.605, Cohen’s *d* = 0.104; delta, *t* (48) = 0.650, *p* = 0.519, Cohen’s *d* = 0.182; and theta, *t* (48) = 0.199, *p* = 0.843, Cohen’s *d* = 0.091], respectively.

**TABLE 3:** The differences between two groups in the whole-brain connectivity wPLI (M ± SD).

	**Experiment group (*N* = 25)**	**Control group (*N* = 25)**
Delta band	0.485 ± 0.022	0.480 ± 0.032
Theta band	0.418 ± 0.012	0.414 ± 0.010
Alpha band	0.353 ± 0.008	0.354 ± 0.011
Beta band	0.358 ± 0.008	0.220 ± 0.008

*p < 0.05.*

**FIGURE 1:**
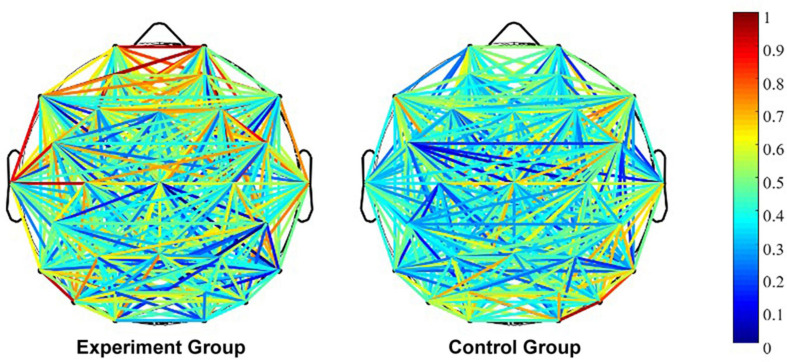
The whole-brain connectivity on beta band. Note: wPLI values presented in line colors.

**FIGURE 2:**
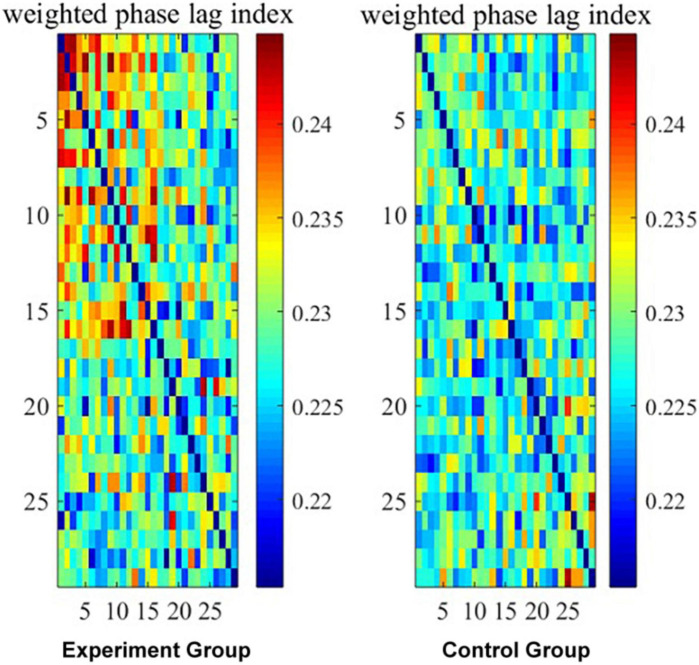
The wPLI interaction metrics on beta band. Note: the wPLI values presented in color blocks.

**FIGURE 3:**
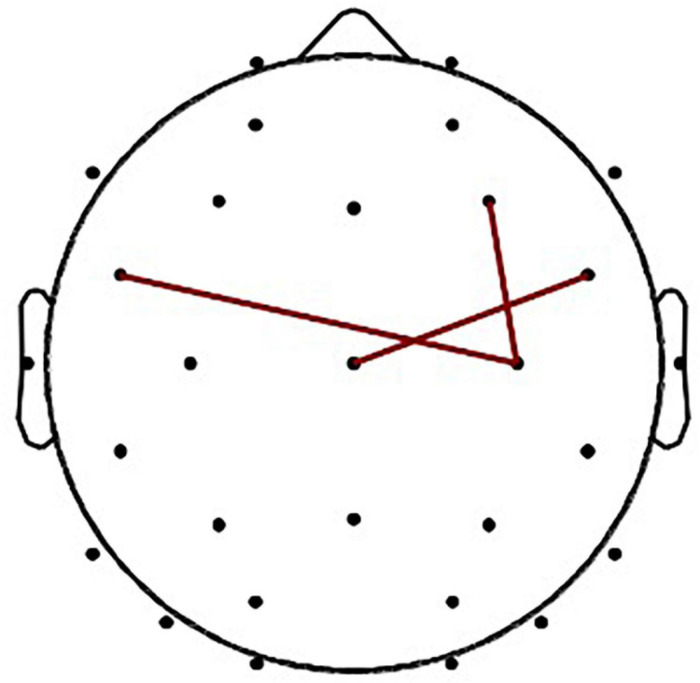
The wPLI in Experiment Group (interaction: F4–C4, FC5–C4, FC6–Cz).

On beta band, whole-brain connectivity is presented in Figure 1, the wPLI interaction metrics is presented in Figure 2, and the significant interaction dynamics (F4–C4, FC5–C4, FC6–Cz) are presented in Figure 3.

Moreover, the study compared wPLI performances between the two groups and found out that the FC of the experimental group was significantly stronger than that of the control group in frontal brain regions in F4–C4 comparison [*t* (48) = 3.818, *p* = 0.0002, Cohen’s *d* = 1.103], FC5–C4 comparison [*t* (48) = 3.701, *p* = 0.0003, Cohen’s *d* = 1.079], and FC6–Cz comparison [*t* (48) = 3.636, *p* = 0.0003, Cohen’s *d* = 1.056], as shown in Table 4.

**TABLE 4:** The significantly higher wPLI value in the experimental group than that in the control group on beta band (M ± SD).

	**Experiment group (*N* = 25)**	**Control group (*N* = 25)**
F4-C4	0.243 ± 0.021	0.222 ± 0.018
FC5-C4	0.236 ± 0.015	0.217 ± 0.020
FC6-Cz	0.243 ± 0.018	0.223 ± 0.020

## Conclusion

The present study incorporated the cognitive neuroscience experiment, with the EEG measures to record brain signals in the news-reading process, and FC method and CSD to analyze FC of EEG signals in different oscillations to observe the influence of two different materials (emotional texts vs. neutral texts) on cognitive processing of individuals, indicating that emotional discourses of news content in the posttruth era could affect the cognitive activities of the audiences.

Electroencephalogram results with δ, θ, α, and β bands revealed that news with emotionalized expression caused a stronger cortical brain activity, consuming more attention resources but fewer cognitive resources (beta oscillations), which may further impede rational thinking of the audiences. More importantly, also on the beta band, it was showed that the brain FC of the emotional news reading group was stronger than that of the control group. This robust connectivity appeared in the central frontal region, consistent with the findings of previous studies (Aydin et al., 2018), and mainly in the right brain hemisphere.

The cross brain regional FC of the beta wave was enhanced, which is related to early emotional processing rather than sustained status (Knyazev et al., 2016), and so individuals may locate more attention resources into emotion-related task processing, and less cognitive resources for content-related tasks, which would lead to the weakening of deep processing, showing the PSD in every band to be significantly reduced, especially the theta band, related to working memory, and the delta band related to complex cognitive tasks (Harmony et al., 1996; Gevins et al., 1997; Krause et al., 2000; Deiber et al., 2007).

On top of that, the synchronous activity on beta band represents an implicit mechanism of perceptual emotion regulation, which may be related to the stress for negative emotion (Wyczesany et al., 2018a, b). The emotional discourses in the experiment, particularly negative emotions in the news text, have functioned as a stressor and elicited the brain regions in the right hemisphere to regulate these emotional threats automatically. In this condition, a massive amount of cognitive resources have been consumed in the emotion regulation process rather than in deep thinking and rational processing of news content, also in accordance with the typical performance of the individuals of the posttruth era (Higgins, 2016; Gewin et al., 2017; Rochlin, 2017).

## Discussion

Belief echoes in the public sphere (Thorson, 2016) states that in the long term, the attitude of the audiences would maintain even if opposite pieces of truth were provided. In the posttruth era, emotions and bias integrate with worldviews and have created “an alternative epistemology that does not conform to conventional standards of evidentiary support” (Lewandowsky et al., 2017). Emotions affect our cognitive processing of information, thus making a difference to our judgment and cognitive strategies (Forgas, 1995, 2010; Milyavsky et al., 2019). The more exposed we are to negative emotional materials, the more time and energy we squander in distracting tasks to deal with them (Isen, 1987). It is concluded that the emotion triggers a priming effect, and emotional expression serves as the noise of communication. Thus, the era of posttruth discourse prioritizes emotion over facts, be it GM food news or other kinds of information.

In summary, in the posttruth era, the news context with emotionalized expression could easily activate cortical activity and connectivity, and attract the attention of the audiences, explaining the phenomenon that conveying information and reporting with emotionalized expression would be easier in the media channels. Compared with the neutral and objective discourses, emotional content is equipped with subjective standpoints, ready to arouse the interest of the audiences when it is consistent with their presumed values, beliefs, and attitudes. Meanwhile, the study has found that audiences may dedicate much more cognition resources to handling those with emotionalized expression with little attention to the actual content or fact itself. In other words, the audience would prefer superficially to stay in the emotions while the emotionalized expression hinders possible deeper consideration of the truth. With the principle of news objectivity unprecedentedly challenged, news content rendered with emotions and opinions induces the truth to be no longer fact-based or objective, one of the critical problems faced by news production as well as the receivers in the age of posttruth.

## Data Availability Statement

The raw data supporting the conclusions of this article will be made available by the authors, without undue reservation.

## Ethics Statement

The studies involving human participants were reviewed and approved by Ethics Committee at the School of Journalism and Communication, Beijing Normal University. The patients/participants provided their written informed consent to participate in this study.

## Author Contributions

YY, LX, and GY participated in the design of the study. YY performed the conceptualization, wrote, and edited the manuscript. LX performed the data analysis and virtualization and wrote the experiment draft. YY and LX conducted the experiment. GY performed the funding acquisition. All authors read and approved the final manuscript.

## Conflict of Interest

The authors declare that the research was conducted in the absence of any commercial or financial relationships that could be construed as a potential conflict of interest.

## Publisher’s Note

All claims expressed in this article are solely those of the authors and do not necessarily represent those of their affiliated organizations, or those of the publisher, the editors and the reviewers. Any product that may be evaluated in this article, or claim that may be made by its manufacturer, is not guaranteed or endorsed by the publisher.
